# A Novel Approach to Assessing and Treating Musculoskeletal-Mediated Atypical Abdominal Pain: A Case Study

**DOI:** 10.7759/cureus.12359

**Published:** 2020-12-29

**Authors:** Alexandra Scozzafava, David P Newman, Aimee N Jacobs, Joshua Sorge, Eric A Elster

**Affiliations:** 1 Physical Therapy Department, Irwin Army Community Hospital, Fort Riley, USA; 2 Interdisciplinary Pain Management Center, Tripler Army Medical Center, Honolulu, USA; 3 Physical Therapy Department, Tripler Army Medical Center, Honolulu, USA; 4 Department of Surgery, Uniformed Services University of the Health Sciences, Bethesda, USA

**Keywords:** chronic abdominal pain, rib, physical medicine and rehabilitation

## Abstract

Abdominal pain is a common and functionally limiting complaint within the healthcare system linked to impaired quality of life and increased health care utilization. This chief complaint is associated with an extensive differential diagnosis leading to high utilization of diagnostic testing, increased healthcare cost, and delayed access to care. In patients presenting with acute or chronic abdominal pain, musculoskeletal pain often requires expensive testing, thereby delaying definitive care. An improved triage process is warranted. Performing a musculoskeletal examination to determine if pain patterns can be mechanically reproduced at the site of origin, or remote to the site of pain, warrants referral to a musculoskeletal specialist. In our young and healthy population, once the musculoskeletal mediated abdominal pain origin is determined, we see significant success in the application of a treatment approach consisting of manipulative therapy, exercise, and instrument-assisted soft tissue mobilization. A multimodal treatment approach for musculoskeletal-mediated abdominal pain has not been previously described. This case study outlines a novel management approach for musculoskeletal-mediated abdominal pain and provides an alternative diagnostic technique, when implemented early in the evaluation and management process of atypical abdominal pain, that improves the quality of life.

## Introduction

Abdominal pain is a common complaint in patients accessing the healthcare system. Approximately 43% of patients presenting to primary care clinics report a chief complaint of abdominal pain [1]. This complaint is functionally limiting and can be associated with impaired quality of life and increased healthcare utilization [2].

Given the non-specific nature and variety of symptoms associated with abdominal pain, a wide differential diagnosis must be considered. Structural gastrointestinal disorders, such as gastroesophageal reflux disease (GERD), peptic ulcer disease, gallbladder pathology, and pancreatitis, in addition to functional disorders associated with abdominal pain including functional dyspepsia, irritable bowel syndrome, and functional abdominal pain syndrome, should be included in the differential. While it is recommended that diagnostic testing be limited and tailored to the clinical features, symptom severity, and response to prior therapy, in many cases, a more thorough and invasive series of tests is performed [2]. In cases of superior quadrant abdominal pain or epigastric pain, an extensive diagnostic workup can include blood tests, endoscopy, and or diagnostic radiographic imaging to include; abdominal radiographs, right upper quadrant ultrasound, upper gastrointestinal series, abdominal computerized tomography (CT) scan, magnetic resonance imaging (MRI), and nuclear medicine hepatobiliary studies. These tests can be costly, invasive, and in some cases, inconclusive. Complaints of epigastric pain may also indicate the need for a cardiac workup leading to further diagnostic testing.

Determining the actual etiology of abdominal pain is very difficult as pain location and characteristics are not always predictive of pain relief. In a sample of 43 consecutive patients with non-specific abdominal pain referred to a Pain Management Clinic from Gastroenterology, only 35% reported long-term relief [3]. Treatment options consistently performed include trigger point injections for myofascial pain, local nerve blocks for neuropathic pain, and oral medications to include non-steroidal, selective serotonin reuptake inhibitors, anti-epileptics, antidepressants, and narcotics. Pain management may not be an effective first-line consultative alternative since these programs emphasize decreasing opioid utilization and optimizing function rather than pain reduction [4].

An under-recognized origin of abdominal pain is the musculoskeletal system, which is typically not addressed as a possible source for pain until the results of expensive and invasive diagnostic testing prove negative [3]. Screening for neuromusculoskeletal abdominal pain can be performed quickly as the pain is most likely triggered by active movement of the involved body region or a combination of palpation and abdominal wall contraction in the case of abdominal cutaneous nerve entrapment syndrome (ACNES) [5,6]. Potential pain generators include the abdominal wall, thoracic spine, rib dysfunction/slipping rib syndrome, fascia, and psoas muscle group [7,8].

In cases of abdominal pain without a definitive diagnosis from diagnostic abdominal testing, referral to a musculoskeletal specialist (i.e., physical therapist, chiropractor, or osteopath) may be warranted in order to perform a low-risk manual evaluation to determine a possible musculoskeletal origin. This assessment can be done during the gastroenterology work-up to reduce time to diagnosis, costs, and potentially unnecessary tests. The musculoskeletal assessment should include provocation testing of the spine and ribs to determine a potential musculoskeletal pain generator [9]. Portions of the physical examination to include a thoracic range of motion (ROM), spinal joint mobility, and rib provocation testing can be done in the primary care setting or the emergency department to assist in their workup and referral management. The purpose of this case study is to describe the application of a multimodal musculoskeletal treatment approach for a patient with chronic atypical abdominal pain and propose diagnostic and treatment recommendations that may serve to decrease the use of potentially unnecessary, costly, and invasive procedures while expediting access to effective care.

## Case presentation

A 34-year-old male patient presented with a 17-month history of intermittent left-sided upper abdominal and epigastric pain. The pain occurred one day following running, lasting three days. The pain was described as aching and stabbing in nature and radiated to the left flank, intermittently ranged in severity from 3/10 to 6/10 on the visual analog scale when present, and affected his ability to participate in recreational as well as physical activities.

Symptom onset was correlated to ingesting a heavy meal consisting of biscuits and gravy with reported abdominal pain and sweats requiring urgent care. In the emergency department (ED), evaluation included a negative abdominal CT and he was discharged on antibiotics. The patient continued to have intermittent symptoms and self-managed by removing meats from his diet and intermittently taking dicyclomine and polyethylene glycol 3350. 

Ten months later, the patient was seen for continued epigastric and left upper quadrant pain occurring randomly and not related to meals. The patient was subsequently referred to as gastroenterology. Upon examination, the pain was not provoked with abdominal auscultation. Murphy’s sign was negative. The workup included an assessment of H pylori to rule out peptic ulcer disease and an endoscope to assess for evidence of candidiasis or obstruction. Red flag symptoms to include anemia, blood in the stool, and weight loss were not present. Labs to include a complete blood count and comprehensive metabolic panel were negative. The working diagnosis was gastritis, and he was treated with pantoprazole 40 mg daily for two months. The patient followed up with gastroenterology as the pain worsened after running four miles and intense workouts. CT of the abdomen and pelvis with contrast, abdominal MRI, magnetic resonance cholangiopancreatography, MRI enterography, and nuclear medicine hepatobiliary studies were all normal. The patient was instructed to follow-up with his primary care provider as the examination did not indicate a gastrointestinal source of pain. 

The patient did report a history of left-sided chest pain in 2017 that did precede his initial complaint of left side abdominal pain. During that period, the patient underwent a cardiology workup to include 12-lead EKG, laboratory studies, and a chest X-ray that were normal. The patient was seen by the Cardiology Clinic and wore an event monitor for two-weeks. No abnormal rhythms were noted, and the patient was discharged with a diagnosis of non-cardiac chest pain. An echocardiogram showed a mildly thickened left ventricle with a good ejection fraction of 60-65% and no dyskinesia or hyperkinesia. The patient was again seen in 2019 with a left-sided chest and tingling into the left arm and back similar to that in 2017. A 12-lead EKG was performed as well as a hemoglobin A1C panel and Lipid screen. Follow-on workup included an exercise treadmill test that was normal.

After 17-months of recalcitrant left upper quadrant pain, the patient was referred to the Interdisciplinary Pain Management Clinic (IPMC). The patient’s primary goal was diagnostic clarity. His secondary goal was to run without pain.

Physical examination

**Figure 1:**
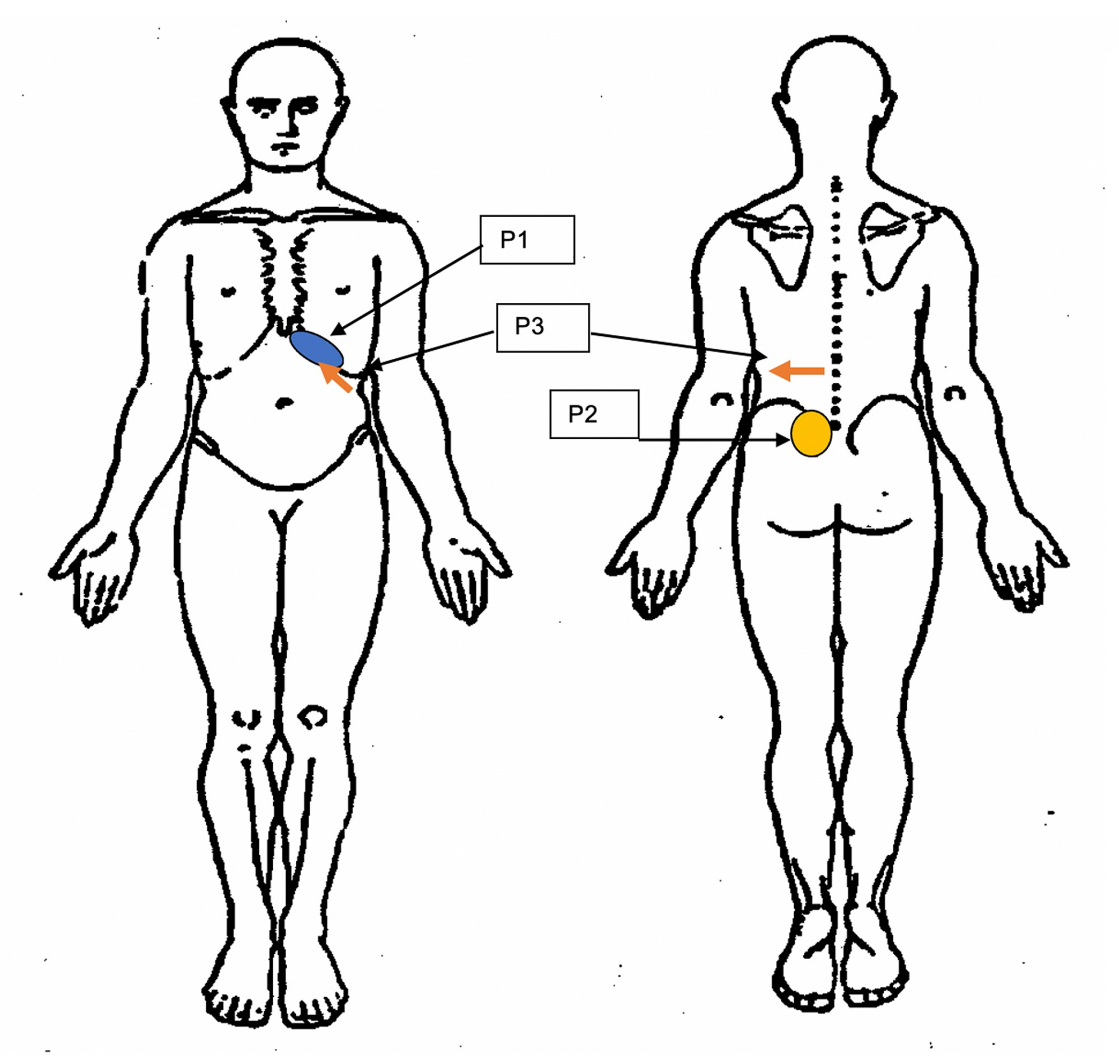
Body chart depicting the patient's pain complaints. P1: pain reported at his initial presentation, P2: pain reported at the second clinic visit, P3: pain reported at the third clinic visit.

Physical evaluation at the IPMC revealed that the patient’s pain was localized to the abdomen just below the costochondral cartilage and extending to a point midway between the umbilicus and axillary line the day following a run (Figure 1). Deep palpation of the area did not reproduce pain. Palpation in conjunction with contraction of the abdominals (Carnett test) did not reproduce symptoms. Pressure applied to the area of the abdominal cutaneous nerve on the left side did not reproduce his pain. Thoracic ROM was assessed in standing. No pain was reproduced with motion along the cardinal planes but the combined thoracic extension and side bending to the right did reproduce his abdominal pain.

Motion palpation testing

**Figure 2:**
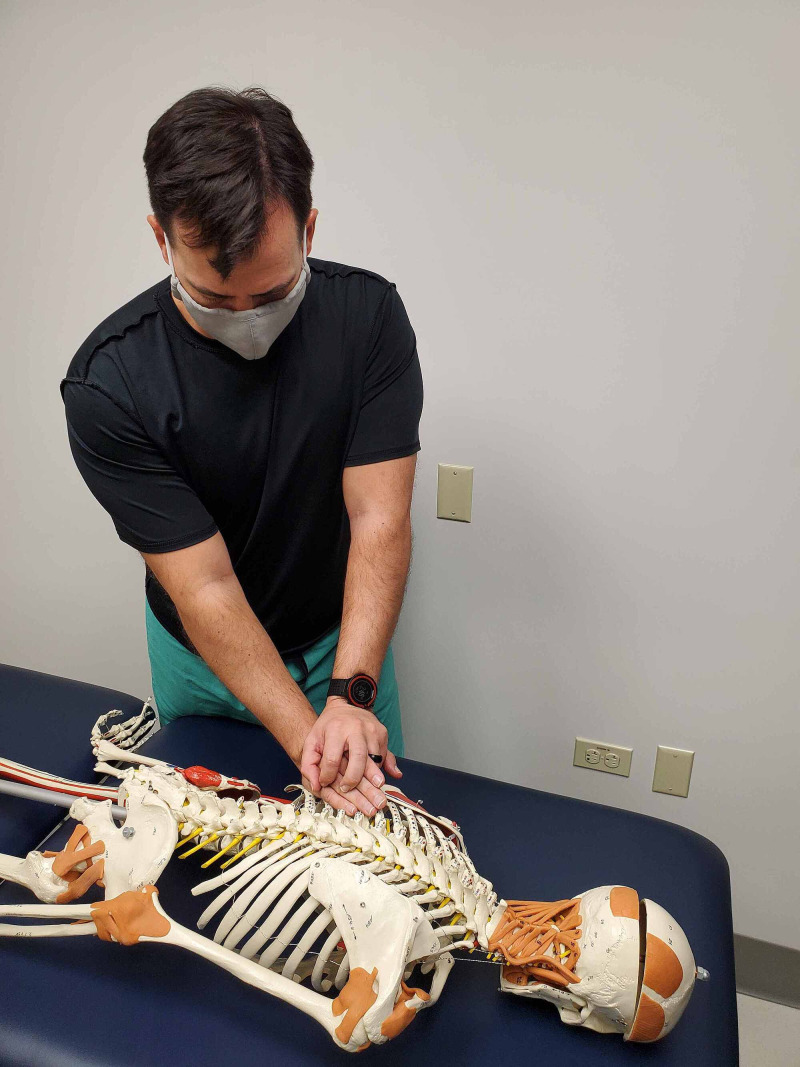
Rib spring maneuver applied to the 11th rib – hand placement on a skeleton. (Photograph: Sorge JA. Rib Spring Maneuver applied to the 11th rib – Hand placement on a skeleton. Reproduced with permission from the author; 2020).

**Figure 3:**
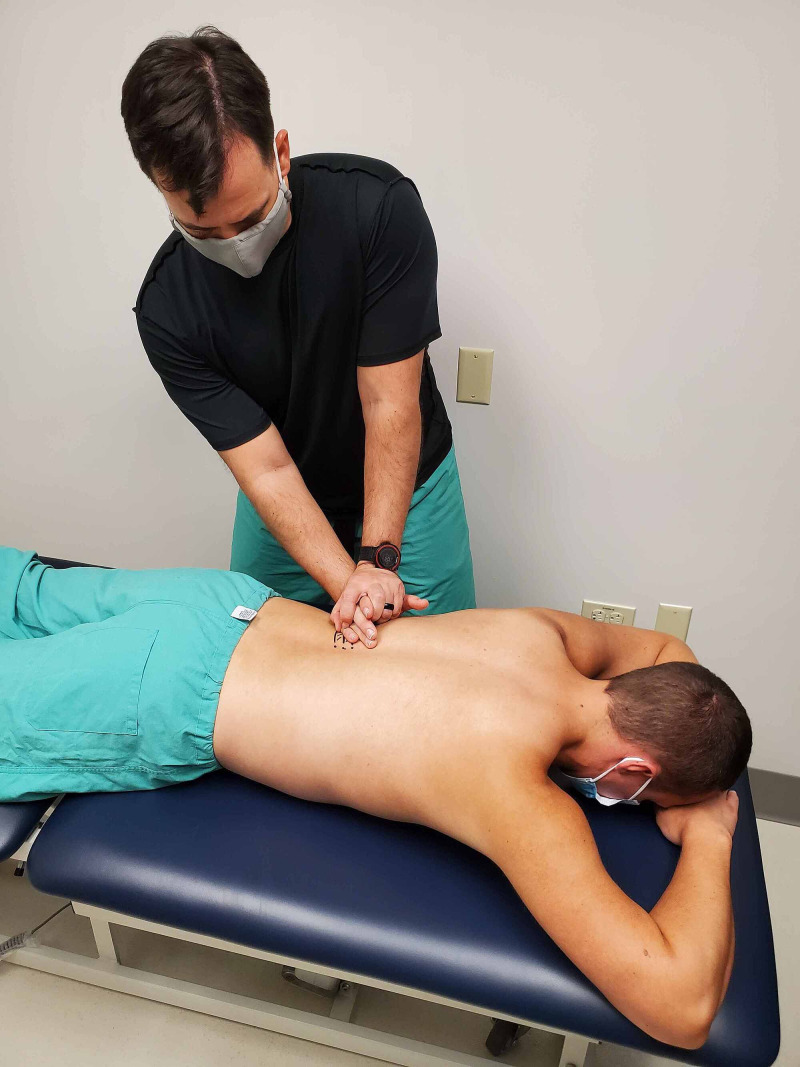
Rib spring maneuver applied to the 11th rib – a technique applied to a simulated patient. (Photograph: Sorge JA. Rib Spring Maneuver applied to the 11th rib – Technique applied to a simulated patient. Reproduced with permission from the author; 2020).

While in prone, provocation testing includes several maneuvers with the goal of identifying biomechanical faults causing or contributing to the patient’s pain complaints. A rib spring maneuver is performed by pressing down on the left 11th rib angle in an anterior direction and then quickly releasing pressure so that the rib springs back up (Figures 2 and 3). A positive test is indicated by a reproduction of the patient’s pain as in this case study patient.

**Figure 4:**
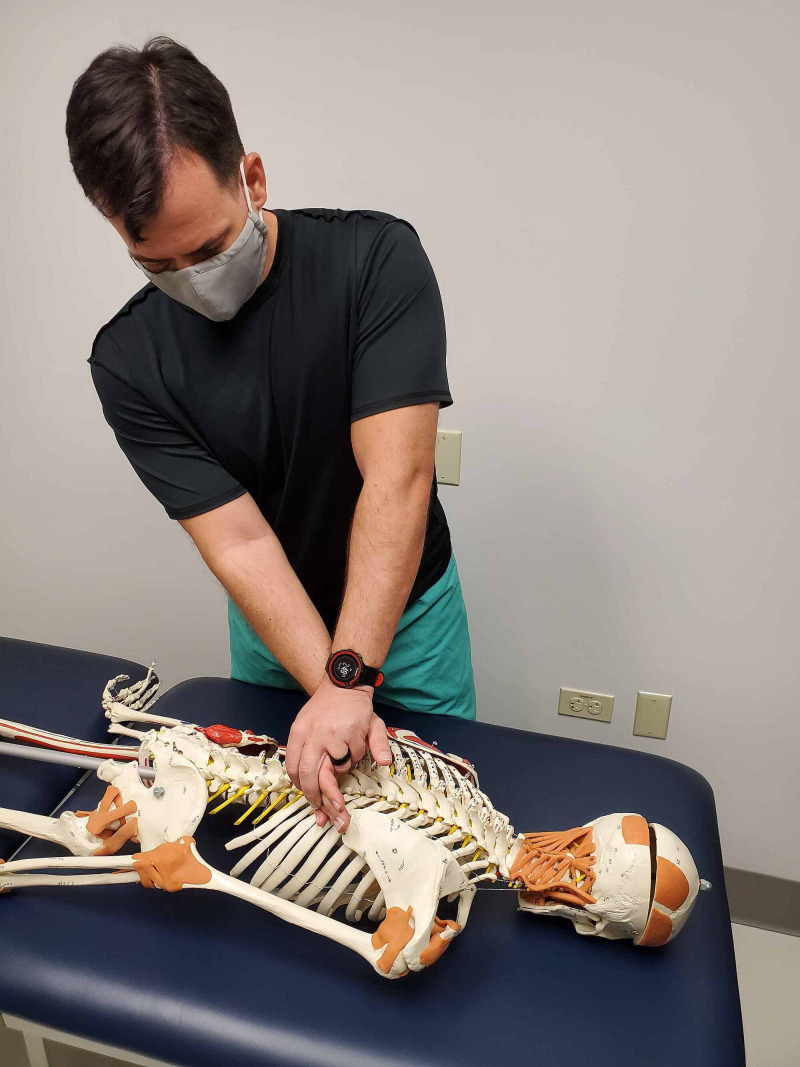
Passive accessory intervertebral motion testing at T10 – hand placement on a skeleton. (Photograph: Sorge JA. Passive accessory intervertebral motion testing at T10 – Hand placement on a skeleton. Reproduced with permission from the author; 2020).

**Figure 5:**
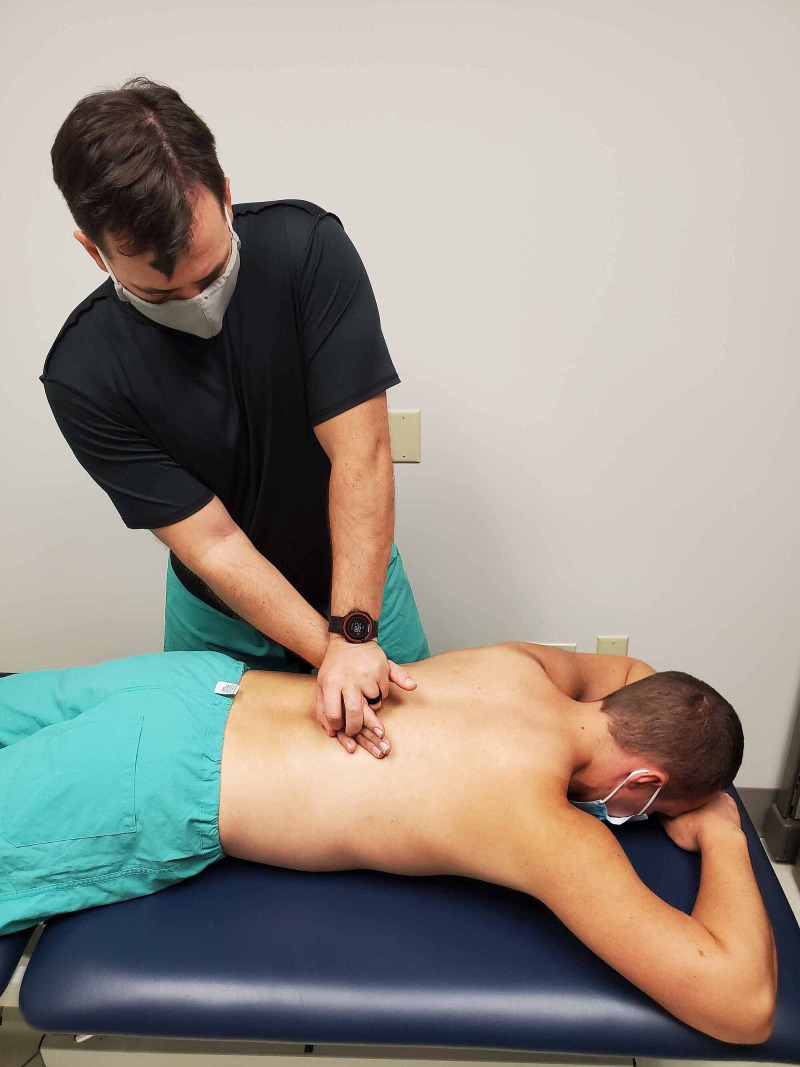
Passive accessory intervertebral motion testing at T10 - a technique applied to a simulated patient. (Photograph: Sorge JA. Passive accessory intervertebral motion testing at T10 – Technique applied to a simulated patient. Reproduced with permission from the author; 2020).

The passive accessory intervertebral motion testing was performed in prone to the T1 through T12 spinous processes. The pressure is applied to each of the spinous processes assessing for any hypomobility or pain reproduction (Figures 4 and 5). Commonly employed by physical therapists, this technique is considered to be valid in assessing spinal segmental hypomobility [10]. Intra-rater reliability is good and inter-rater reliability is moderate when assessing joint mobility and stiffness in the thoracic spine and ribs [10]. These maneuvers can be performed by primary care or emergency room providers. The restricted motion was assessed at T9 through T11 without pain reproduction.

Strength and flexibility assessment

Flexibility and strength assessments were made in supine. The treating physical therapist surmised that adaptive tissue tightening or muscular imbalance may have occurred due to the chronicity of the patient's complaint. The pectoralis minor and latissimus were tight on the left side compared to the right. The pectoralis major strength was assessed with manual resistance and no chest or abdominal pain was reproduced. Having the patient performing ten sit-ups assessed abdominal strength based on physical requirements in the U.S. Marine Corps. No pain was reproduced during the performance.

Intervention

**Figure 6:**
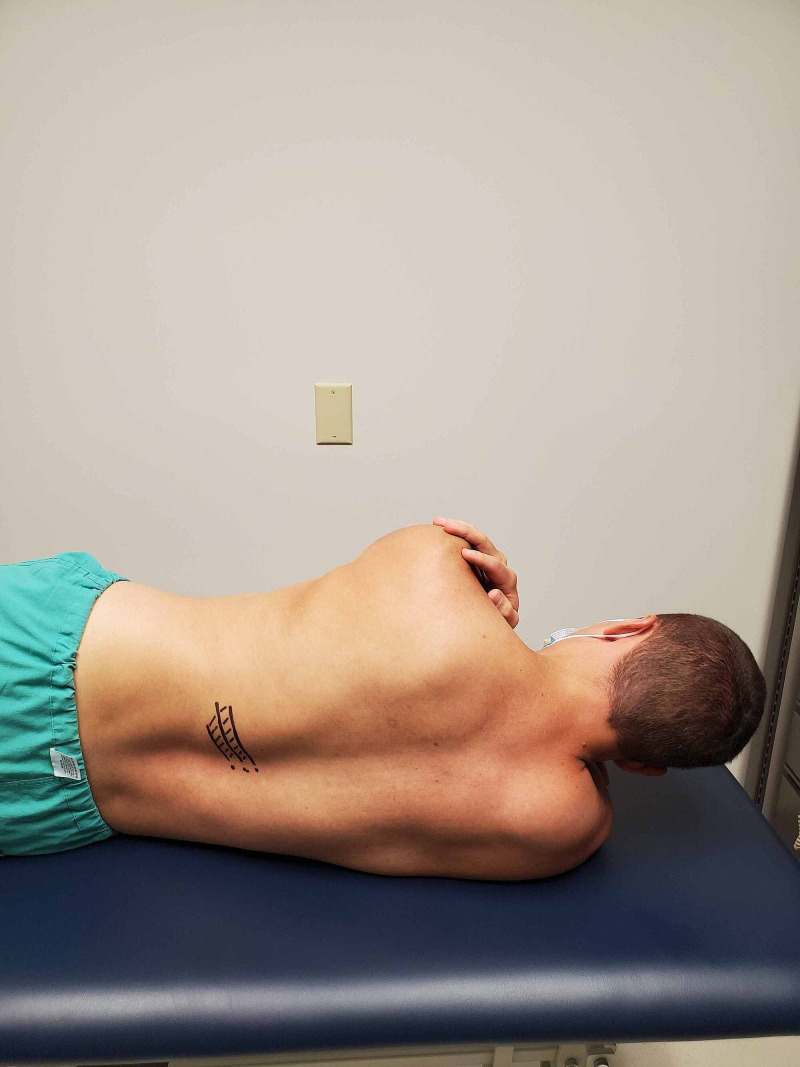
Patient positioning – with the simulated patient in side lying, the location of the hypomobile rib is identified. In this example, the 10th through the 12th ribs are drawn with intercostal muscles visualized by striped lines. (Photograph: Sorge JA. Patient positioning – 11th rib osteopathic manipulation technique. Reproduced with permission from the author; 2020).

**Figure 7:**
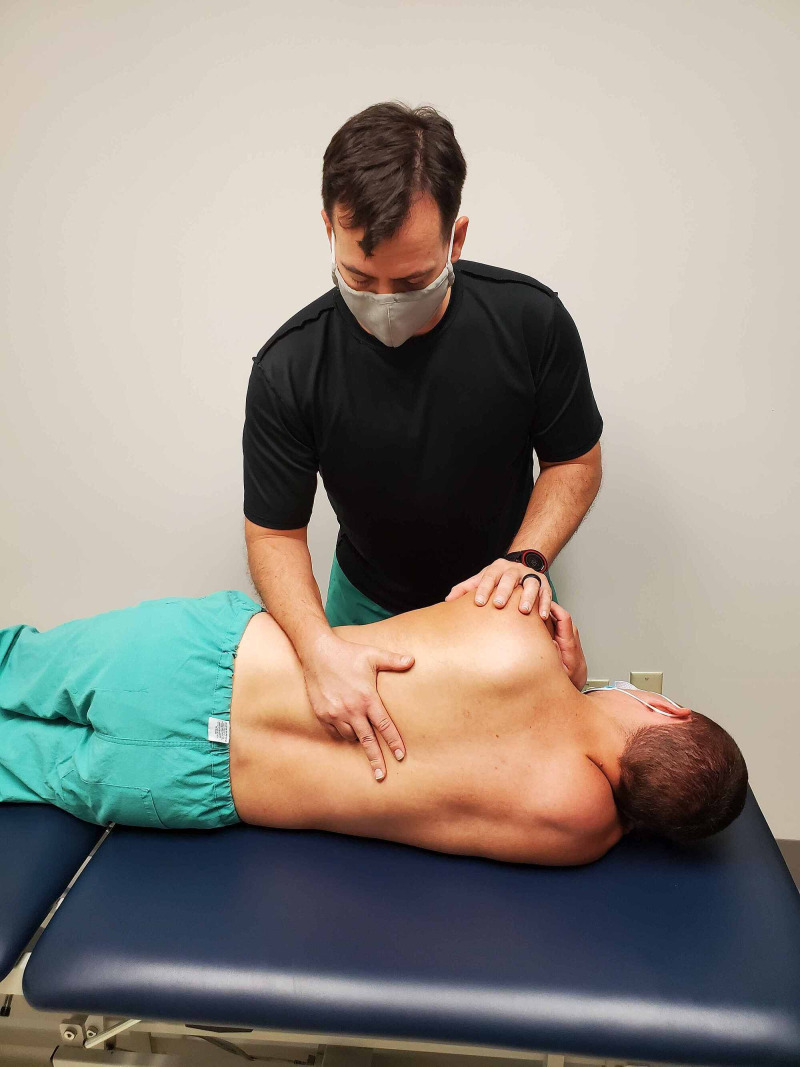
Hand positioning – the provider’s first metacarpal phalangeal joint of the palpating hand is placed over the 11th rib at the costotransverse joint and slid inferiorly to induce a posterior rotation to the rib. (Photograph: Sorge JA. Hand positioning – 11th rib osteopathic manipulation technique. Reproduced with permission from the author; 2020).

**Figure 8:**
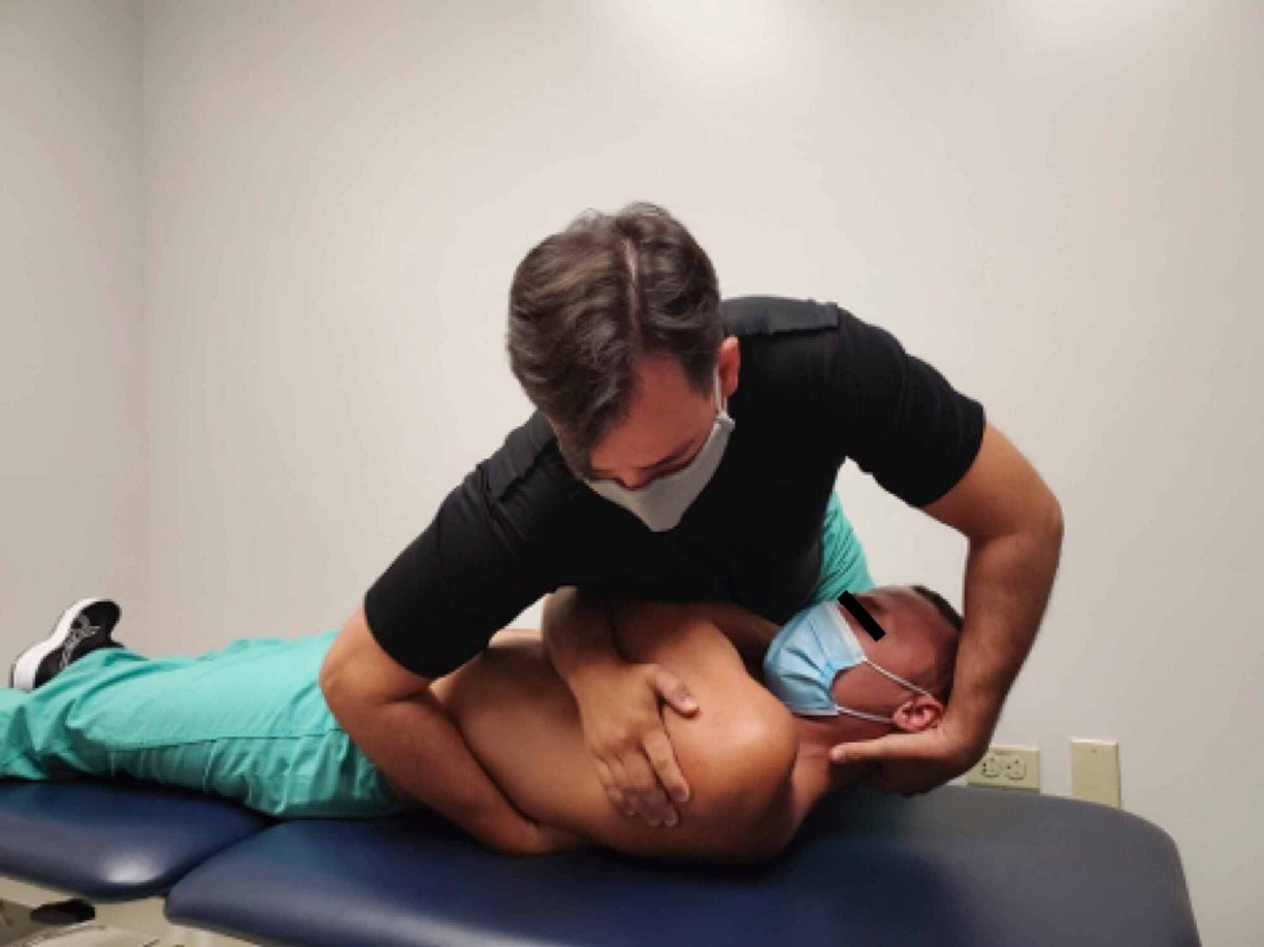
Manipulation technique – the provider moves the simulated patient into supine. While the simulated patient exhales, the provider imparts a high velocity, low amplitude force through the simulated patient’s arms toward the examiner’s hand. (Photograph: Sorge JA. Manipulation technique - Thrust maneuver to the 11th Rib. Reproduced with permission from the author; 2020).

The patient was treated upon the initial evaluation with osteopathic manipulation techniques (OMT; Table 1). OMT has been shown to be effective in the treatment of rib dysfunction [11]. A posterior rotation force was applied to the left 11th rib with audible cavitation (Figures 6-8). Upon active thoracic ROM, the patient’s abdominal pain was not reproduced with combined extension and rotation to the right side.

**Table 1:** Overview of interventions applied and patient response per visit. PSIS: posterior superior iliac crest, LBP: low back pain, IPMC: Interdisciplinary Pain Management Clinic, PAVIM: passive accessory intervertebral movement, OMT: osteopathic manipulation technique, SIJ: sacroiliac joint, ROM: range of motion.

Visit	Patient	Objective Findings	Intervention	Patient Response
1	Pain localized to the area of abdomen below costochondral cartilage and extending to a point midway between the umbilicus and axillary line (P1) the day following a run.	P1 reproduced by combined thoracic extension and side bending to the right; 11^th^ rib spring maneuver. Restricted movement T9 through T11 with PAIVMs without pain provocation. Tight left pectoralis minor and latissimus muscles.	11^th^ rib OMT.	P1 not reproduced with combined thoracic extension and side bending to the right.
2	No P1 pain after jogging. Pain at left PSIS (P2).	Elevated iliac crest on the left side. (+) Thigh thrust. Tight left piriformis and iliopsoas muscles.	SIJ OMT. Deep tissue mobilization of the piriformis muscle. Active release of the iliopsoas muscle. Prescribed lumbopelvic strengthening program.	No P1 after treatment. P2 is not reproduced with thigh thrust maneuver.
3	P1 pain returned. No P2 pain. Constant pain along 10^th^ and 11^th^ rib origin and radiating anteriorly to 11^th^ rib tip (P3).	P3 reproduced by rotating trunk fully to left. 11^th^ rib spring maneuver. T10/11 segmental hypomobility.	11^th^ rib OMT.	No P1 and P3 with provocation or ROM testing.
4	No P1 pain after jogging 4 miles P1 and P3 pain after jogging 7 miles.	Pain along 10^th^ rib angle with thoracic rotation to left P3 reproduced by 10^th^ rib spring maneuver pain along the costochondral cartilage from 10^th^ rib tip to the sternum.	10^th^ rib OMT directional cupping to costochondral cartilage.	No P1 and P3 with provocation or ROM testing.
5-7	Residual pain at P1 and P3 after activity.	The pain reported with pressure applied to the costochondral cartilage.	Directional cupping to costochondral cartilage. Active release of pectoralis minor and latissimus muscles.	No pain at P1 or P3 with provocation or ROM testing.
8	Recurrence of P1, P3, and new LBP after surfing two hours. P1 after jogging 5 miles.	P1 reproduced by deep pressure to the attachment of abdominals to the costochondral cartilage on the left side.	Directional cupping to costochondral cartilage.	No pain reported.
9	No pain with jogging. Mild tenderness at P1 with pressure.	P1 reproduced by deep pressure to the attachment of abdominals to the costochondral cartilage on the left side.	Directional cupping to costochondral cartilage.	No pain reported.
10	No pain reported.	No findings of the physical examination.	Discharged from the IPMC.	

No other treatment was performed at that time. The patient was asked to go jogging later in the day and assess for changes in pain along the abdomen the following day. The patient was asked to contact the clinic telephonically with the results.

Upon telephonic follow-up, the patient reported that he did jog that day, and went snorkeling and bike riding the following day. The patient did not experience any left-sided upper quadrant pain, but he did note some flank pain as well as pain along the 11th rib and minor sternal pain. The patient wanted to go jogging again prior to his next clinic follow-up to determine if there was an actual treatment effect versus placebo effect from OMT.

At the second clinic visit two days later, the patient reported no abdominal or epigastric pain despite jogging the day previously. The patient did report pain at the left posterior superior iliac crest (PSIS; Figure 1). A physical examination was performed. There was no abdominal pain reported with thoracic ROM or pain along the 11th rib with provocation testing. There was an asymmetry in the patient’s pelvic landmarks with his PSIS and iliac crest elevated on the left side as compared to the right suggesting sacroiliac joint (SIJ) dysfunction. This was confirmed by performing a thigh thrust. During the thigh thrust, the patient is supine with hip and knee flexed. The examiner cups the sacrum with one hand and applies force axially through the knee providing a shear force to the SIJ. A positive test is indicated by joint hypomobility, when compared to the opposite side, and reproduction in pain. There was also tightness noted along with the left piriformis and iliopsoas when assessed in prone.

Given signs and symptoms consistent with concomitant SIJ dysfunction, the patient was treated with a combination of SIJ OMT, deep tissue mobilization of the piriformis, and active release of the iliopsoas as described previously by Newman et al. [12]. The patient was instructed in a lumbopelvic strengthening program directed at the hamstrings, hip adductors, and abdominals to be done every other day, as well as stretching of the piriformis and iliopsoas muscles daily. The patient was asked to follow-up in one week.

At the third visit, the patient’s flank pain pattern returned. The patient ran three miles three times since the last visit. The patient did not report any abdominal or sacroiliac joint, SIJ pain either during or after jogging. He did report a constant pain along the 10th and 11th rib origin that radiated anteriorly to the 11th rib tip, which started the previous day (Figure 1). Rotating the trunk fully to the left side reproduced this pain. Provocation testing revealed a reproduction of pain along the 11th rib and T10/11 segmental hypomobility. OMT to the 11th rib on the left side completely resolved his pain. As his abdominal pain goal had been met, his functional goal was revised to be able to jog seven miles without flank, back, or costochondral pain in four weeks.

**Figure 9:**
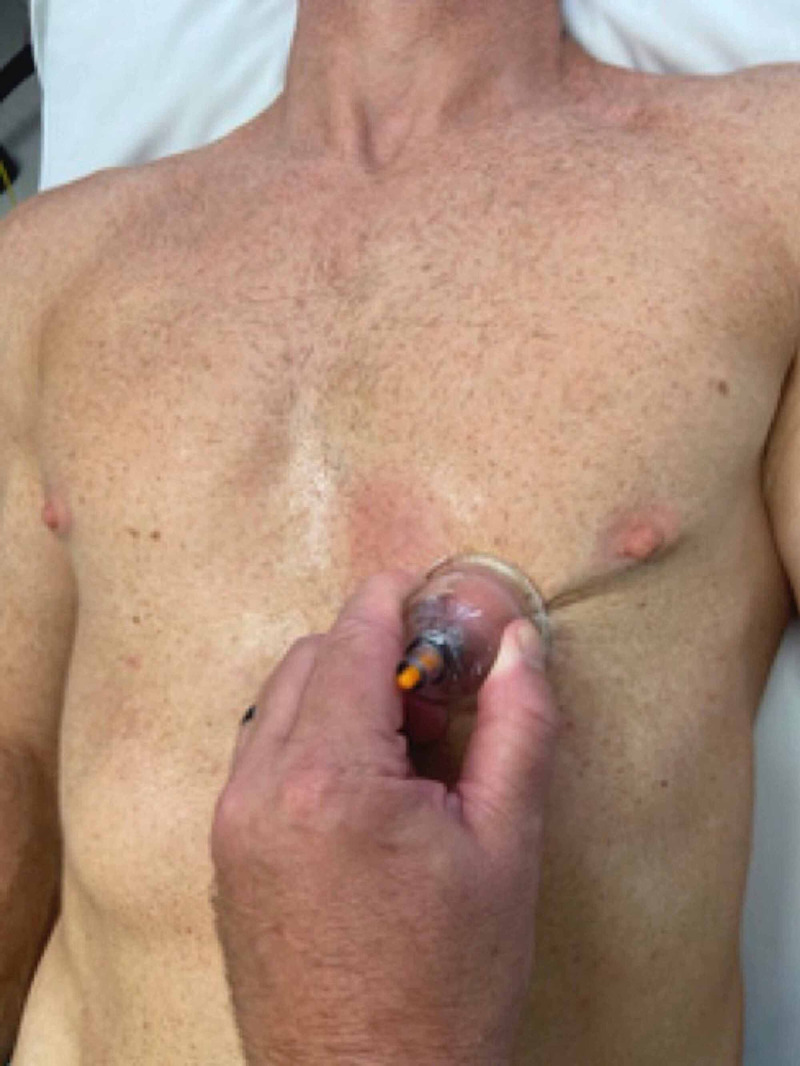
Directional cupping technique – the cup with applied to the sternum and suction applied. The cup was then moved manually by the provider along the costochondral cartilage and superior margin of the abdominal attachment to the 10th rib tip and then back to the sternum. This was performed for a period of 30 seconds. (Photograph: Newman DP. Directional cupping technique. Reproduced with permission from the author; 2020).

Upon follow-up at the fourth visit, the patient reported no abdominal or flank pain with jogging four miles. When he increased the distance to seven miles, the flank pain returned as well as pain along the costochondral cartilage extending to the distal sternum. This pain lasted two days and then subsequently resolved. Physical examination revealed pain along the 10th rib angle with thoracic rotation to the left side. Provocation testing to the 10th rib was positive for pain reproduction, but not at the 11th and 12th ribs. No pain was reproduced with deep palpation of the abdomen; however, there was pain along the costochondral cartilage extending from the 10th rib tip to the sternum. Following OMT to the 10th rib, instrument-assisted deep tissue mobilization was applied to the skin above the costochondral cartilage utilizing a directional (moving) cupping technique (Figure 9) [13].

At the fifth through seventh visits, the patient reported residual pain along the 10th rib and costochondral cartilage after activity lasting several hours. The patient had been increasing his activity level to include working out, running, and surfing. Treatment directed at the mobilization of the myofascial tissue along the costochondral cartilage was repeated. In addition, active release techniques were applied to the pectoralis minor and latissimus muscles to determine if tissue tightness contributed to rib mobility. After the performance of these techniques, the patient reported no pain with repeat provocation or ROM testing. The patient would continue to follow-up weekly for reassessment until his functional goals were met.

Upon follow-up at the eighth visit, the patient reported a recurrence in pain along the abdomen, flank, and low back following surfing for two hours. The pain remained constant all day and night, thereby affecting his sleep. All of his symptoms resolved by the next day. The patient subsequently went jogging five miles the previous day and experienced tightness along the costochondral cartilage immediately afterward that has remained constant until this appointment. The patient rated his pain level as a 3/10 on a visual analog scale. The physical assessment did not demonstrate any segmental or rib hypomobility. The pain was reproduced with deep pressure applied to the attachment of the abdominals to the costochondral cartilage on the left side. The directional cupping treatment was repeated in the painful area. No pain was noted after treatment.

Approximately five weeks after starting care, the patient came in for the eighth and ninth visits, the patient reported no flank, back, or costochondral pain with jogging seven miles. The patient continued to have mild tenderness along the costochondral cartilage with deep pressure; therefore, the directional cupping technique was employed to optimize tissue mobility in order to reduce the chance of recurrence. The following week, the patient returned for his tenth and final visit. The patient performed a series of intensive workouts to include jogging and surfing daily. There was no return of his pain. The patient was subsequently discharged from the IPMC.

## Discussion

In patients presenting with chronic, non-specific abdominal wall pain, it can be difficult to accurately identify a specific diagnosis or pain generator given a very long differential to include visceral, functional, abdominal wall, and neuromusculoskeletal conditions. The neuromusculoskeletal abdominal wall differential encompasses thoracic dysfunction, thoracic disc herniation, intercostal nerve entrapment, rib dysfunction, slipping rib syndrome, and myofascial pain syndrome, which overlap with ACNES. Potential neuroanatomic mechanisms for referred abdominal pain from the spine include specific nerve root entrapment such as a thoracic disc herniation or thoracic radiculopathy [14], secondary nerve root compression from muscle spasm, intercostal nerve compression anywhere along its course, and referred pain from the vertebral joints [15].

Conservative evaluation and management of rib-mediated abdominal pain can be provided through a combination of manipulative therapy, exercise, and myofascial mobilization designed to correct biomechanical faults remote to the area of pain and supporting myofascial structures at the area of pain. This case study demonstrates the successful application of such a multimodal treatment program and identifies potential factors that if not addressed may lead to less than optimal outcomes.

At the initial presentation of abdominal pain in the ED or primary care setting, a neuromusculoskeletal examination may be cursory, not made at all, or deferred to another specialty. According to Bharucha et al. (2016), abdominal pain is treated as a functional gastroenterological disorder until proven otherwise (i.e., rule out GERD, peptic ulcer, functional dyspepsia, or functional abdominal pain) [2]. If not functional or organic, then the abdominal wall would be next on the differential [6,16]. Given the high costs and delayed definitive care while ruling out an extensive differential [17], we argue that rib and joint provocation testing described above can be included at the initial presentation. These maneuvers are easy to perform, low risk, and can provide better options for referral upon discharge from the primary care clinic or ED.

In addition to detailed patient history and suspicion of the chest wall as the pain generator, a clinical examination includes the Carnett test. The Carnett test has a diagnostic accuracy of 97% for abdominal wall pain [18]. If the Carnett test is positive in patients with acute abdominal pain [16], the following diagnoses may be considered: iatrogenic peripheral nerve injuries if a history of surgery, abdominal cutaneous nerve entrapment, hernias, myofascial pain syndromes, rib tip syndrome, and abdominal pain from a spinal origin. Gallegos and Hobsley developed an assessment algorithm that considered neuromusculoskeletal diagnoses for abdominal pain based on the response to the Carnett test; however, this approach is cumbersome and takes significant time and cost to complete [16]. This algorithm did include an examination of the spine and ribs for referral patterns.

In our case, the Carnett test was non-contributory which may be attributed to the chronicity of symptoms and delayed evaluation. Upon presentation to the IPMC, the patient did not report any specific abdominal pain. The patient reported that pain occurred the day after jogging and persisted for one to two days. The Carnett test may have been more useful during the acute period when he presented initially to the ED or his primary care physician; therefore, care should be taken when weighting the differential exclusively on abdominal tenderness when assessing chronic abdominal pain.

Other published diagnostic and treatment algorithms lack specific referral to a musculoskeletal specialist. Bharucha et al. developed one for chronic abdominal pain [2]. They did include abdominal wall pain as a working diagnosis following a positive Carnett test; however, there were no referral recommendations. Vaghef-Davari et al. presented 10 practice algorithms for acute abdominal pain presenting to primary care or ED physicians [17]. These algorithms were developed based on the site of pain or patient demographics. Of the seven based on pain location, none of them recommended referral to a musculoskeletal specialist or proposed musculoskeletal treatment options.

Successful functional outcomes achieved by the patient in this case study may be attributed to a systematic approach that leveraged continuous evaluation, response to treatment, and identification of biomechanical faults remote to the actual area of pain. As the patient’s symptoms were only reproduced following jogging, the immediate response to the initial rib mobilization was not expected. Instead, the patient was asked to go jogging and then self-assess. The response was indeed successful; however, caution was needed to ensure long-term benefit. While the patient did report no pain with jogging along the left upper quadrant, there was pain consistent with a left-sided sacroiliac joint dysfunction. While it is difficult to determine if this outcome was or was not contributory to the patient's rib and myofascial dysfunction, it was treated with OMT and exercise described in an evidenced-based algorithmic treatment approach [12].

Follow-up assessment unmasked the ribs and spinal segmental hypomobility as the main pain generators consistent with a phenomenon called regional interdependence [9]. In this model, functional impairments remote to the area of pain and that may be considered unrelated to the actual diagnosis actually do contribute to the problem. This phenomenon would explain why the expensive and exhaustive diagnostic testing directed at the abdomen was ineffective. Consideration of the patient’s history of non-cardiac chest pain that preceded the abdominal pain may have been the key to early, definite treatment especially if the problem was rib mediated and the patient had been referred to a musculoskeletal specialist [19].

Further evaluation at each follow-up visit revealed other compensatory changes that contributed to the patient’s abdominal pain. At the fourth visit, the patient noted pain extending from the 10th rib to the sternum. The 11th rib was no longer an issue. At later visits, potential adaptive changes to the fascia and muscles connecting to the ribs and costochondral cartilage needed to be addressed to prevent a recurrence. Manipulative therapy in isolation may not have resulted in optimal results. Exercise and myofascial mobilization are important treatment adjuncts [17]. Myofascial mobilization is an effective treatment modality in treating abdominal wall trigger points [20].

Although our case report describes the inherent difficulty in identifying the actual cause of abdominal pain and offers a novel musculoskeletal-oriented treatment approach, there are limitations. A single case report may not be generalizable to a wider population. A larger case-controlled study or prospective cohort study is warranted. Second, the patient did have several risk factors to include a several-year history of alcohol abuse and chest pain which did warrant initial gastroenterology and cardiopulmonary testing. Finally, the patient was well-conditioned and wanted to return to work that involved heavy lifting, physical stress, and running. These factors may be incongruent in patients with chronic abdominal pain that has been recalcitrant to multiple courses of ineffective treatment.

## Conclusions

Abdominal pain is a common diagnosis seen in the ED and primary care settings. Patients may undergo a comprehensive and often costly diagnostic workup that may ultimately be inconclusive. Once structural or functional gastrointestinal disorders are ruled out, a musculoskeletal origin may be considered, but definitive care may not be possible due to compensatory changes of the muscular or skeletal structures. Our case represents a patient with an atypical case of abdominal pain whose referral to the IPMC team was delayed by 17 months due to the extensive diagnostic work-up and poor response to accepted standard treatment options for abdominal pain. After being treated with a novel, multimodal treatment program consisting of physical therapy osteopathic manipulation, soft tissue mobilization, and exercise, the patient had full resolution of his abdominal pain. This case demonstrates the potential benefit of including low-risk musculoskeletal assessment techniques in the ED or primary care triage process for the evaluation of abdominal pain that may improve specialty care utilization and reduce healthcare costs.

## References

[1] Vandvik PO, Kristensen P, Aabakken L, Farup PG (2004). Abdominal complaints in general practice. Scand J Prim Health Care.

[2] Bharucha AE, Chakraborty S, Sletten CD (2016). Common functional gastroenterological disorders associated with abdominal pain. Mayo Clin Proc.

[3] McGarrity TJ, Peters DJ, Thompson C, McGarrity SJ (2000). Outcome of patient with chronic abdominal pain referred to chronic pain clinic. Am J Gastroenterol.

[4] Rome JD, Townsend CO, Bruce BK, Sletten CD, Luedtke CA, Hodgson JE (2004). Chronic noncancer pain rehabilitation with opioid withdrawal: Comparison of treatment outcomes based on opioid use status at admission. Mayo Clin Proc.

[5] Harding G, Yelland M (2007). Back, chest and abdominal pain- Is it spinal referred pain?. Aust Fam Physician.

[6] Lindsetmo R, Stulberg J (2009). Chronic abdominal wall pain - a diagnostic challenge for the surgeon. Am J Surg.

[7] Rodeghero JR, Denninger TR, Ross MD (2013). Abdominal pain in physical therapy practice: 3 patient cases. JOSPT.

[8] Kamboj KK, Hoversten P, Oxentenko AS (2019). Chronic abdominal wall pain: a common yet overlooked etiology of chronic abdominal pain. Mayo Clin Proc.

[9] Sueki DG, Cleland JA, Wainner RS (2013). A regional interdependence model of musculoskeletal dysfunction: research, mechanisms, and clinical implications. J Man Manip Ther.

[10] Heiderscheit B, Boissonnault W (2008). Reliability of joint and pain assessment of the thoracic spine and rib cage in asymptomatic individuals. J Man Manip Ther.

[11] Kasten KM, Lewis DD (2020). High-velocity, low-amplitude management of posterior rib somatic dysfunction. J Am Osteopath Assoc.

[12] Newman DP, McLean BC, Scozzafava AM (2020). Evaluation and management of sacroiliac dysfunction utilizing an evidence-based algorithmic approach: a case study. Cureus.

[13] Murray D, Clarkson C (2019). Effects of moving cupping therapy on hip and knee range of movement and knee flexion power: a preliminary investigation. J Man Manip Ther.

[14] Ishii M, Nishimura Y, Hara M (2020). Thoracic disc herniation manifesting as abdominal pain along associated with thoracic radiculopathy. NMC Case Rep J.

[15] Ashby EC (1977). Abdominal pain of spinal origin. Value of intercostal block. Ann R Coll Surg Engl.

[16] Gallegos NC, Hobsley M (1990). Abdominal wall pain: an alternative diagnosis. Br J Surg.

[17] Vaghef-Davari F, Ahmadi-Amoli H, Sharifi A, Teymouri F, Paprouschi N (2020). Approach to acute abdominal pain: practical algorithms. Adv J Emerg Med.

[18] Greenbaum DS, Greenbaum RB, Joseph JG, Natale JE (1994). Chronic abdominal wall pain. Diagnostic validity and costs. Dig Dis Sci.

[19] Newman DP, Jansen Jansen, BJ BJ, Scozzafava A, Smith R, McLean BC (2020). Rib mediated non-cardiac chest pain: a case report. Cureus.

[20] Musculino JE (2013). Abdominal wall trigger point case study. J Bodywork Movement Therapy.

